# Comprehensive Analysis of Cytomegalovirus pp65 Antigen-Specific CD8^+^ T Cell Responses According to Human Leukocyte Antigen Class I Allotypes and Intraindividual Dominance

**DOI:** 10.3389/fimmu.2017.01591

**Published:** 2017-11-21

**Authors:** Seung-Joo Hyun, Hyun-Jung Sohn, Hyun-Joo Lee, Seon-Duk Lee, Sueon Kim, Dae-Hee Sohn, Cheol-Hwa Hong, Haeyoun Choi, Hyun-Il Cho, Tai-Gyu Kim

**Affiliations:** ^1^Department of Microbiology, College of Medicine, The Catholic University of Korea, Seoul, South Korea; ^2^Catholic Hematopoietic Stem Cell Bank, College of Medicine, The Catholic University of Korea, Seoul, South Korea; ^3^College of Medicine, Cancer Research Institute, The Catholic University of Korea, Seoul, South Korea

**Keywords:** human leukocyte antigen class I, cytomegalovirus pp65, CD8^+^ T cell, immunodominant pattern, human leukocyte antigen restriction, preferential usage

## Abstract

To define whether individual human leukocyte antigen (HLA) class I allotypes are used preferentially in human cytomegalovirus (CMV)-specific cytotoxic T lymphocyte responses, CD8^+^ T cell responses restricted by up to six HLA class I allotypes in an individual were measured in parallel using K562-based artificial antigen-presenting cells expressing both CMV pp65 antigen and one of 32 HLA class I allotypes (7 HLA-A, 14 HLA-B, and 11 HLA-C) present in 50 healthy Korean donors. The CD8^+^ T cell responses to pp65 in the HLA-C allotypes were lower than responses to those in HLA-A and -B allotypes and there was no difference between the HLA-A and HLA-B loci. HLA-A*02:01, -B*07:02, and -C*08:01 showed the highest magnitude and frequency of immune responses to pp65 at each HLA class I locus. However, HLA-A*02:07, -B*59:01, -B*58:01, -B*15:11, -C*03:02, and -C*02:02 did not show any immune responses. Although each individual has up to six different HLA allotypes, 46% of the donors showed one allotype, 24% showed two allotypes, and 2% showed three allotypes that responded to pp65. Interestingly, the frequencies of HLA-A alleles were significantly correlated with the positivity of specific allotypes. Our results demonstrate that specific HLA class I allotypes are preferentially used in the CD8^+^ T cell immune response to pp65 and that a hierarchy among HLA class I allotypes is present in an individual.

## Introduction

The fraction of a population infected with cytomegalovirus (CMV) depends on the socioeconomic status of the population. CMV has a prevalence of 60–70% in industrialized countries, whereas in emerging countries this rate is nearly 100% (1). After primary infection, which frequently occurs during early childhood (1), CMV establishes lifelong latency under control of the immune system because of the numerous virus evasion strategies that interfere with the host immune response at many levels (2). Human leukocyte antigen (HLA)-restricted cytotoxic T lymphocytes (CTLs) are involved in the early immune response and are important defense mechanism in CMV infections (3). Transplant-related morbidity and mortality in CMV-seropositive patients are increased despite highly effective prevention of CMV disease after allogeneic T cell-depleted stem cell transplantation (4). Furthermore, CMV is one of the common congenitally transmitted viruses capable of causing severe illness in newborns (5). Protection from CMV disease in immune-compromised hosts has been correlated with recovery of host virus-specific CD8^+^ T cell responses (6). Protective immunity can be successfully transferred by infusion of donor-derived CMV-specific CD8^+^ CTL clones (7, 8). Assessment of clonal diversity of T cell responses against human CMV, a major cause of morbidity in immune-depressed patients, provides important insights into the molecular basis of T cell immunodominance and has clinical implications for immune monitoring and immunotherapy of CMV infections (9).

Identification of the CTL epitopes derived from CMV is valuable for monitoring antiviral immunity and for *ex vivo* generation of antiviral CTLs for possible application in adaptive immunotherapy (10). The lower matrix protein 65 (pp65), a structural protein that is abundant throughout CMV infection, is an important subject of CMV research. It is widely accepted as the immune-dominant target of the CD8^+^ T cell response against CMV (11). Analysis of the fine specificity of pp65-specific CTL showed that some donors have a highly focused response recognizing only a single peptide, whereas others recognize multiple peptides throughout the pp65 gene product (12). However, previously identified CTL epitopes derived from pp65 protein were limited to traditionally well-studied HLA class I allotypes such as HLA-B*07 (13). Thus, relatively little is known about epitopes presented on infrequently observed allotypes (14).

The high level of polymorphism within the HLA region may provide an advantage in host defense against pathogen mediated by T cells (15). Among the epitopes presented by HLA allotypes, certain peptides known to have immunodominance are more frequently recognized than others, which is suggested to be related to peptide-binding repertoires of different sizes, affinities, and immunogenicities (16, 17). Immunodominance according to HLA allotypes is variably used to describe either the most frequently detectable response among tested individuals or strongest response within a single individual. Although the factors affecting immunodominance have been studied, immunodominance of HLA allotypes to CMV remain unexplored.

Cytomegalovirus-specific CD8^+^ T cell populations in humans have been studied using *ex vivo* tools, such as major histocompatibility complex class I tetramers and interferon-γ (IFN-γ)-based enzyme-linked immunospot (ELISPOT) assays (18). There is a need for new strategies with improved efficiency and feasibility to detect T cell mediated immune responses on multiple epitopes presented on different HLA allotypes. ELISPOT using pp65-transduced CD40-activated B cells has been used for identifying CTL epitopes presented by various HLA allotypes (10). Epstein–Barr virus (EBV)-specific CD8^+^ T cell responses can be evaluated using autologous dendritic cells transfected with EBV latent membrane protein 1 and latent membrane protein 2A mRNA (19). To comprehensively analyze CD8^+^ T cell responses against the CMV pp65 antigen restricted by a single HLA class I allotype, we conducted ELISPOT assays using an artificial antigen-presenting cell (aAPC) expressing both the pp65 antigen and each HLA class I allotype present in a donor. Our data showed that CD8^+^ T cells responses differed for each HLA allotype, and a specific HLA allotype showed a dominant response, compared with the other HLA allotypes in an individual.

## Materials and Methods

### Donors and Cells

The use of human material was reviewed and approved by Institutional Review Board of the Catholic University of Korea (MC16SNSI0001). Informed consent was obtained according to the Catholic University of Korea. Written informed consent was obtained from all participants involved in this study. Peripheral blood mononuclear cells were collected from 50 healthy Korean donors, using Ficoll-Hypaque (GE Healthcare, Pittsburgh, PA, USA). The average age of the participants was 29.56 ± 3.83 years and consisted of 5 females and 45 males. CD8^+^ T cells were isolated positively using magnetic microbeads (MACS, Miltenyi Biotec, Bergisch Gladbach, Germany) and were cryopreserved until use. HLA typing was carried out at the Catholic Hematopoietic Stem Cell Bank (Seoul, Korea; Table 1).

**Table 1:** Genotypes of HLA class I alleles in 50 healthy Korean donors.

Donor	Age	Sex	HLA-A*	HLA-B*	HLA-C*
HD01	28	M	02:01/33:03	35:01/44:03	04:01/14:03
HD02	30	M	33:03/33:03	44:03/44:03	07:06/14:03
HD03	30	M	02:01/33:03	44:03/54:01	01:02/14:03
HD04	25	M	33:03/33:03	35:01/44:03	04:01/14:03
HD05	30	M	24:02/33:03	07:02/35:01	07:02/08:01
HD06	24	M	02:01/24:02	15:01/59:01	01:02/04:01
HD07	31	M	02:01/11:01	15:01/40:01	03:04/04:01
HD08	35	M	02:01/11:01	15:11/54:01	01:02/03:03
HD09	21	M	24:02/33:03	44:03/51:01	14:02/14:03
HD10	26	M	02:01/24:02	07:02/51:01	07:02/14:02
HD11	34	M	24:02/33:03	07:02/44:03	07:02/14:03
HD12	27	M	11:01/33:03	15:01/44:03	04:01/14:03
HD13	34	F	02:01/33:03	15:01/44:03	04:01/14:03
HD14	33	M	02:01/11:01	15:01/51:01	04:01/14:02
HD15	24	F	02:01/24:02	35:01/54:01	01:02/08:01
HD16	22	M	02:01/24:02	07:02/15:01	01:02/07:02
HD17	29	M	11:01/11:01	15:01/40:01	03:04/04:01
HD18	29	M	02:01/11:01	15:01/15:01	04:01/08:01
HD19	29	M	02:01/11:01	40:01/54:01	01:02/03:04
HD20	29	M	02:01/31:01	35:01/54:01	01:02/03:03
HD21	36	M	31:01/33:03	40:06/44:03	07:06/08:01
HD22	29	F	02:01/33:03	40:06/44:03	03:04/07:06
HD23	31	M	31:01/33:03	35:01/44:03	03:03/14:03
HD24	34	M	02:01/02:01	48:01/54:01	03:04/08:01
HD25	28	M	02:01/02:06	46:01/54:01	01:02/01:02
HD26	28	M	02:01/02:07	27:05/46:01	01:02/01:02
HD27	31	M	02:01/02:06	15:01/15:11	03:03/07:02
HD28	29	M	02:01/02:07	15:11/46:01	01:02/03:03
HD29	31	M	11:01/24:02	15:01/40:01	03:04/04:01
HD30	25	M	11:01/24:02	15:01/59:01	01:02/04:01
HD31	30	M	24:02/24:02	35:01/51:01	03:04/14:02
HD32	27	M	24:02/24:02	40:06/51:01	08:01/14:02
HD33	27	M	02:07/24:02	07:02/46:01	01:02/07:02
HD34	29	M	02:07/11:01	27:05/46:01	01:02/02:02
HD35	28	M	24:02/31:01	07:02/48:01	07:02/08:01
HD36	33	M	24:02/33:03	15:01/58:01	07:02/14:02
HD37	29	M	24:02/33:03	40:01/58:01	03:02/03:04
HD38	34	M	24:02/33:03	40:01/44:03	03:04/14:03
HD39	33	M	24:02/31:01	40:01/51:01	07:02/14:02
HD40	34	M	02:01/11:01	27:05/40:06	01:02/08:01
HD41	37	M	11:01/24:02	27:05/54:01	01:02/02:02
HD42	38	M	11:01/24:02	40:06/48:01	08:01/08:01
HD43	33	M	02:01/24:02	46:01/51:01	01:02/14:02
HD44	29	M	02:06/02:06	48:01/51:01	08:01/14:02
HD45	30	M	02:06/33:03	44:03/48:01	07:06/08:01
HD46	32	M	02:06/33:03	44:03/54:01	01:02/14:03
HD47	29	M	02:06/31:01	15:01/46:01	01:02/03:03
HD48	23	F	31:01/31:01	15:01/51:01	04:01/14:02
HD49	24	F	02:07/24:02	07:02/46:01	01:02/07:02
HD50	27	M	02:07/31:01	07:02/27:05	01:02/07:02

### Establishment of aAPCs

The cDNA of costimulatory molecules (CD83, 4-1BBL, and CD80) was individually cloned into the pCDH lentivirus vector (#CD523A-1; SBI, Palo Alto, CA, USA) and CMV pp65-GFP was cloned into a PiggyBac vector (#PB511B-1; SBI, Palo Alto, CA, USA). For production of lentivirus, 10 µg of a cloned pCDH plasmid and lentivirus packaging plasmids (5 µg pMD2.G and 5 µg psPAX2, cat nos. #12259, #12260; Addgene, Cambridge, MA, USA) were cotransfected into HEK293 cells using lipofectamine reagent (Invitrogen, Carlsbad, CA, USA). Titration of lentivirus was measured by serial dilution and finally transduction was performed with MOI = 1. K562 were cultured in RPMI supplemented with penicillin (100 U/mL), streptomycin (100 U/mL), l-glutamine (2 mM; all from Lonza, Basel, Switzerland), and 10% fetal bovine serum (Life Technologies, Carlsbad, CA, USA). For transduction of costimulatory molecules, 5 × 10^5^ K562 cells/mL were seeded in a 24-well plate. After 24 h, 500 µL lentiviral supernatant and 8 µg/mL polybrene were added to the K562 cell culture. After 48 h, K562 cell were analyzed by flow cytometry (FACS Canto II, BD Biosciences, San Diego, CA, USA). Subsequently, the aAPCs were transfected with PiggyBac-CMV pp65-GFP to produce aAPC-pp65 by the Amaxa Nucleofector™ I (Lonza, Basel, Switzerland) using Amaxa^®^ Cell Line Nucleofector^®^ Kit V (program number T-16), according to the manufacturer’s instructions. Untransfected K562 cells were used as controls. The stable transfectants were positively selected by sorting on a MoFlo Astrios flow cytometer (Beckman Coulter, Indianapolis, IN, USA), using antibody staining.

### Establishment of Transient HLA Class I Expression on aAPCs

Human leukocyte antigen class I (HLA-A; 7, -B; 14, -C; 11) cDNA was individually cloned into the pcDNA 3.1 vector (#V79020; Invitrogen, Carlsbad, CA, USA) and transfected into aAPC and aAPC-pp65 using Amaxa^®^ Cell Line Nucleofector^®^ Kit V (program number T-16), according to the manufacturer’s instructions. To increase the HLA gene expression efficiency, DNA concentrations ranging from 0.1 to 8 µg were tested. HLA class I expression was maintained for 3 days and confirmed by flow cytometry. aAPC and aAPC-pp65 were transfected with six HLA class I allotypes corresponding to the donor HLA type, and immunogenicity was investigated by IFN-γ ELISPOT assay.

### Analysis of aAPCs Phenotype by Flow Cytometry

The expression of HLA class I, costimulatory molecules, and CMV pp65-GFP on aAPCs and aAPC-pp65 was analyzed by flow cytometry. The following antibodies were used in this study: anti-HLA class I (APC; G46-2.6), anti-CD83 (PE; HB15e), and anti-CD80 (PE; L307.4) purchased from BD Biosciences and anti-4-1BBL (PE; 5F4) purchased from BioLegend. Approximately more than 10,000 cells were acquired and the data were analyzed using FLOWJO software (Tree Star, USA).

### IFN-γ ELISPOT Assay

The IFN-γ ELISPOT assay was performed as described previously in literature, with modifications (19). Briefly, autologous CD8^+^ T cells (10^6^ cells) were serially diluted in complete RPMI and 100 µL of each cell suspension was then transferred into the wells, followed by the addition of 200 µL of aAPC and aAPC-pp65 (10^5^ cells) untransfected or transfected with individual HLA-matched single HLA allotype. We performed the experiments only when the expression of each HLA allotype was confirmed by FACS in all tests and more than 50% positive by K562 control (Figure S1 in Supplementary Material). The spot number was counted using an AID ELISPOT Reader System (AID Diagnostika GmbH, Strassberg, Germany).

### Statistical Analysis

For data analysis and visualization, Prism 7.0 software (GraphPad, San Diego, CA, USA) was used. Statistical significance was determined by one-way ANOVA and Pearson’s correlation analysis. The results were obtained from a single experiment on 50 donors. *P*-value ≤ 0.05 was considered significant, as described in figure legends.

## Results

### Optimal Conditions to Detect pp65-Specific CD8^+^ T Cell Responses Restricted by Single HLA Class I Allotype

**Figure 1:**
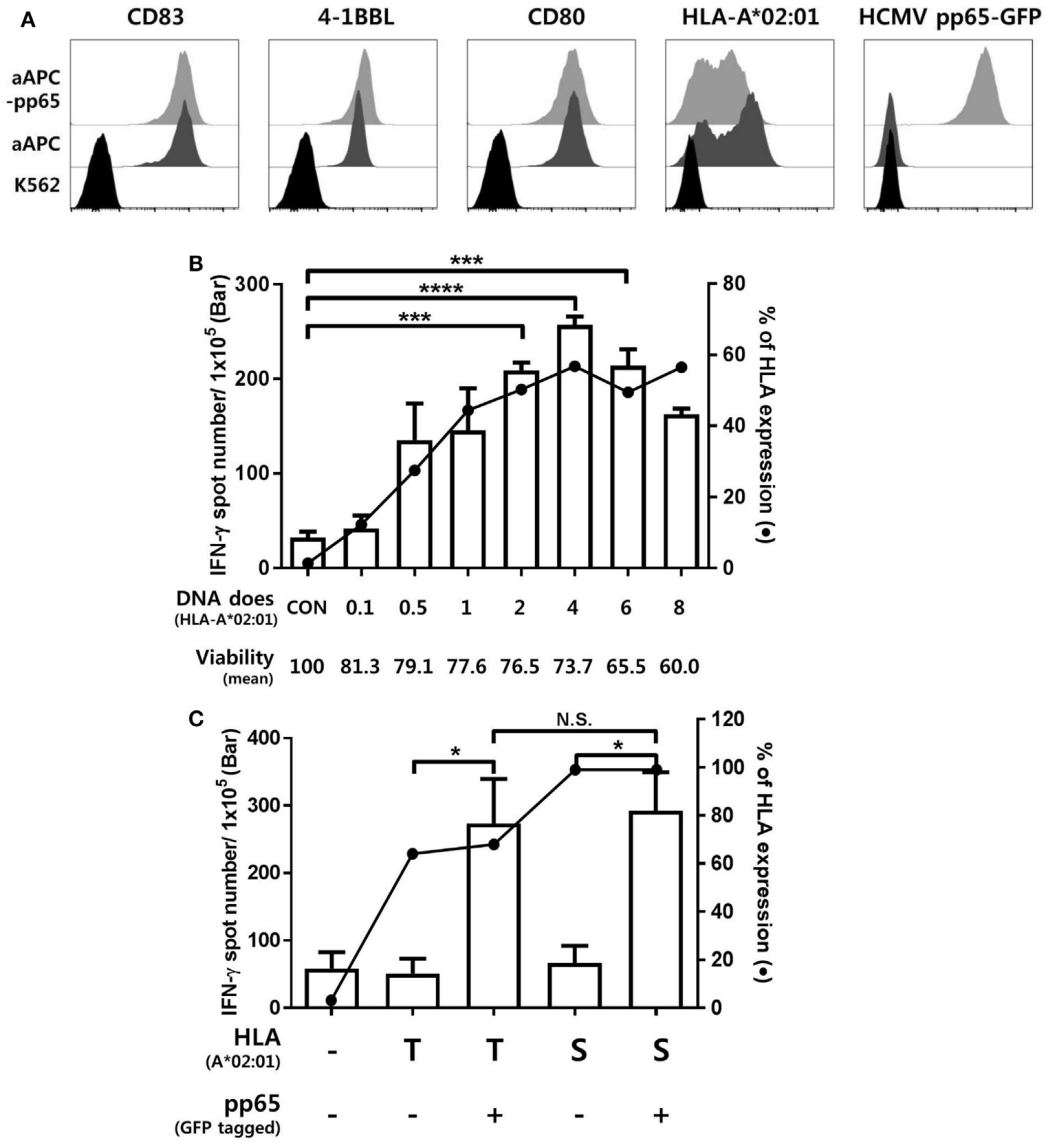
Establishment of an artificial antigen-presenting cell (aAPC) system to detect cytomegalovirus (CMV) pp65-specific CD8^+^ T cell responses restricted by a single human leukocyte antigen (HLA) class I allotype. **(A)** Establishment of aAPCs expressing CD83, 4-1BBL, and CD80, and aAPC-pp65 expressing CMV pp65 from the K562 cell line. To detect the CD8^+^ T cell response to CMV pp65, aAPC-pp65 were transfected with HLA-A*02:01 plasmid DNA. The expression of transferred genes was assessed by flow cytometry. **(B)** Optimization of HLA class I gene transfection to detect CD8^+^ T cell responses. HLA-A*0201 plasmid DNA concentrations ranging from 0.1 to 8 µg were used for transfection in 1 × 10^6^ aAPC-pp65. CD8^+^ T cells showing strong responses to pp65 (HD01) were cocultured with aAPC-pp65 for the interferon-γ (IFN-γ) enzyme-linked immunospot (ELISPOT) assay. **(C)** Comparison of transient transfection with 4 µg of plasmid DNA (T) and stable expression with lentivirus vector (S) of HLA-A*02:01 in stimulating CD8^+^ T cells. CD8^+^ T cells (HD01) were cocultured with aAPC (−) and aAPC-pp65 (+) for IFN-γ ELISPOT assay. Results represent the mean percentage of expression from three independent experiments with SD (bars). *P* values were calculated by 1-way ANOVA (*P < 0.05; **P < 0.01; ***P < 0.001; ****P < 0.0001; N.S., not significant).

K562-derived aAPCs expressing CMV pp65 (aAPC-pp65) were established by transfection with PiggyBac vector expressing CMV pp65 and GFP to detect CMV pp65-specific CD8^+^ T cell responses (Figure S2 in Supplementary Material). The costimulatory molecules CD80, CD83, and 4-1BBL were expressed at similar levels between the aAPCs and aAPC-pp65 coexpressing pp65 and GFP (Figure 1A). To measure CD8^+^ T cell responses restricted to a single allotype among various HLA class I allotypes, it is considered more efficient to transiently express a single HLA gene in aAPCs rather than to establish a stable cell line. The optimal concentration of the HLA-A*02:01 plasmid DNA for transfecting into aAPC-pp65 was determined as 4 µg plasmid DNA per 10^6^ aAPC-pp65 based on the expression of HLA class I molecules and IFN-γ ELISPOT assay using CD8^+^ T cells isolated from the HLA-A*02:01-positive donor HD01, with a strong response to pp65 (Figure 1B). This optimized transfection condition showed T cell stimulation efficiency similar to that in aAPC-pp65 stably expressing HLA-A*02:01 (Figure 1C). Subsequent analysis of various HLA class I genes was performed similarly using appropriate conditions for transient expression (Figure S3 in Supplementary Material).

**Figure 2:**
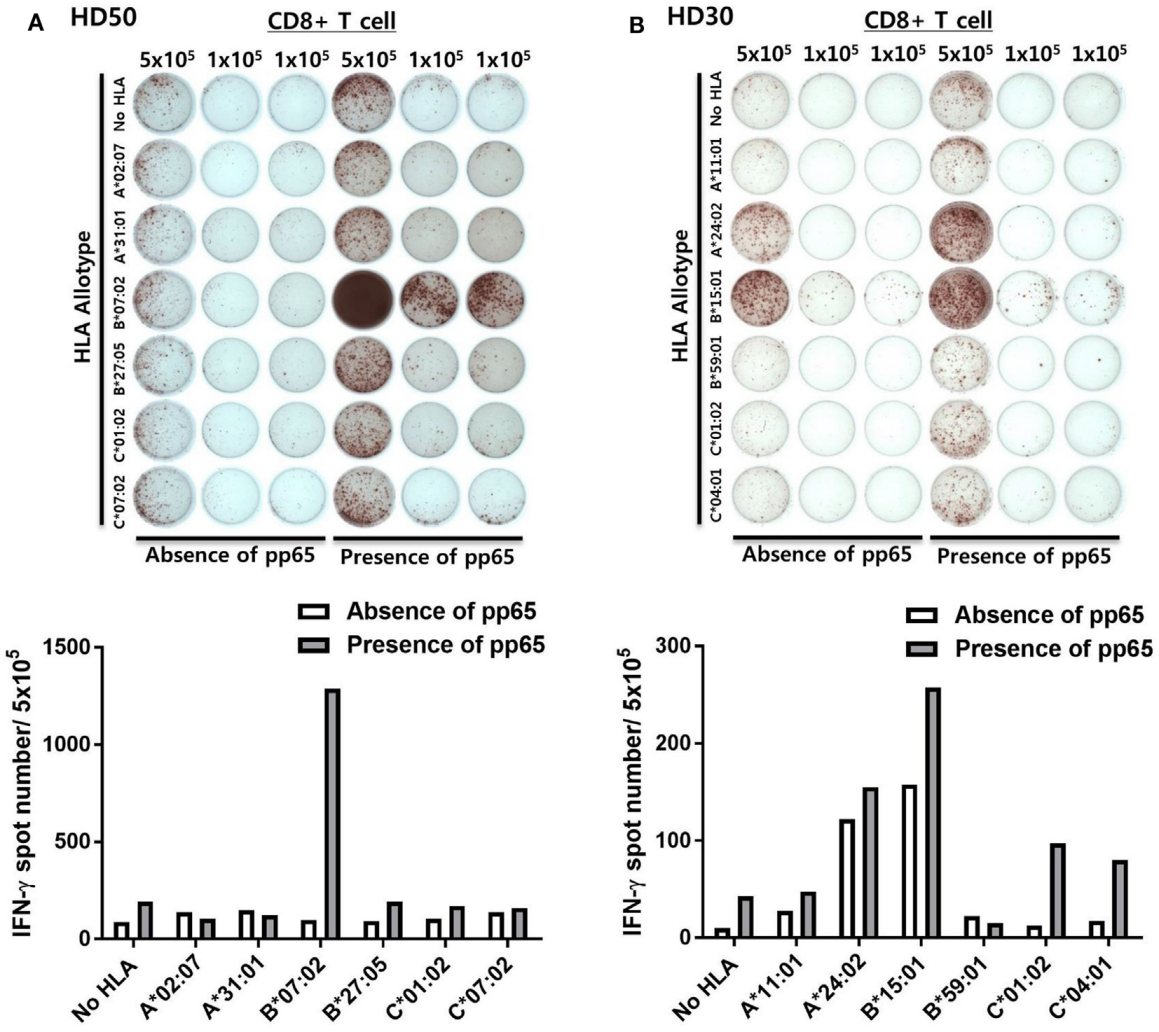
Simultaneous measurement of cytomegalovirus (CMV) pp65 antigen-specific CD8^+^ T cell responses presented by each of the six human leukocyte antigen (HLA) class I allotypes in an individual by enzyme-linked immunospot (ELISPOT). **(A)** Representative ELISPOT assay results. The frequency of CD8^+^ T cells secreting interferon-γ (IFN-γ) was measured using aAPCs (aAPC-HLA) and aAPC-pp65 (aAPC-pp65-HLA) transfected with individual HLA class I allotypes (HLA-A*02:07, -A*31:01, -B*07:02, -B*27:05, -C*01:02, and -C*07:02) of HD50. CD8^+^ T cells were seeded at 5 × 10^5^ per well in one well and at 1 × 10^5^ per well in two wells for each allotype and background groups. The background for aAPCs and aAPC-pp65 was defined as the frequency of IFN-γ secreting CD8^+^ T cells induced by each aAPC without HLA gene transfer. The frequency of IFN-γ secretion in CD8^+^ T cells, excluding background values, is presented. Finally, the frequency of pp65-specific T cells was determined as [(aAPC-pp65-HLA) − (aAPC-pp65)] − [(aAPC-HLA) − (aAPC)]. **(B)** Representative example showing non-specific responses in aAPCs expressing specific HLA allotypes (HLA-A*24:02 and -B*15:01) in HD30.

Figure 2 shows representative ELISPOT results for measuring pp65-specific CD8^+^ T cell responses presented by six HLA class I allotypes in an individual. aAPCs that express a single HLA allotype (aAPC-HLA) and do not express pp65 were used as controls to obtain a background value. Most of the results were obtained with 1 × 10^5^ CD8^+^ T cells per well, with 5 × 10^5^ cells per well used for a low response. In the case of HD50, the frequency of CMV pp65-specific CD8^+^ T cells secreting IFN-γ was high only in HLA-B*07:02, whereas the other allotypes did not differ from controls (Figure 2A). In HD30, HLA-A*24:02 and -B*15:01 showed positive responses with more than 100 IFN-γ spots per 5 × 10^5^ CD8^+^ T cells in both aAPC-pp65 and aAPCs. In general, these HLA-restricted responses were only observed in the presence of pp65, whereas some HLA allotypes showed a positive response even in the absence of the pp65 antigen (aAPC) (Figure 2B). Therefore, the frequency of pp65-specific T cells restricted by each self-HLA allotype was determined as [(aAPC-pp65-HLA) − (aAPC-pp65)] − [(aAPC-HLA) − (aAPC)]. After compensation for background responses by each self-HLA allotype, pp65-specific responses by HLA-C*01:02 and -C*04:01 were higher than that by HLA-A*24:02. In addition, non-specific responses to the self-HLA allotype in the absence of pp65 are frequently observed in certain allotypes and are thought to be responses against K562 minor antigens (Figure S4 in Supplementary Material).

### Dominance According to HLA Class I Allotypes

**Figure 3:**
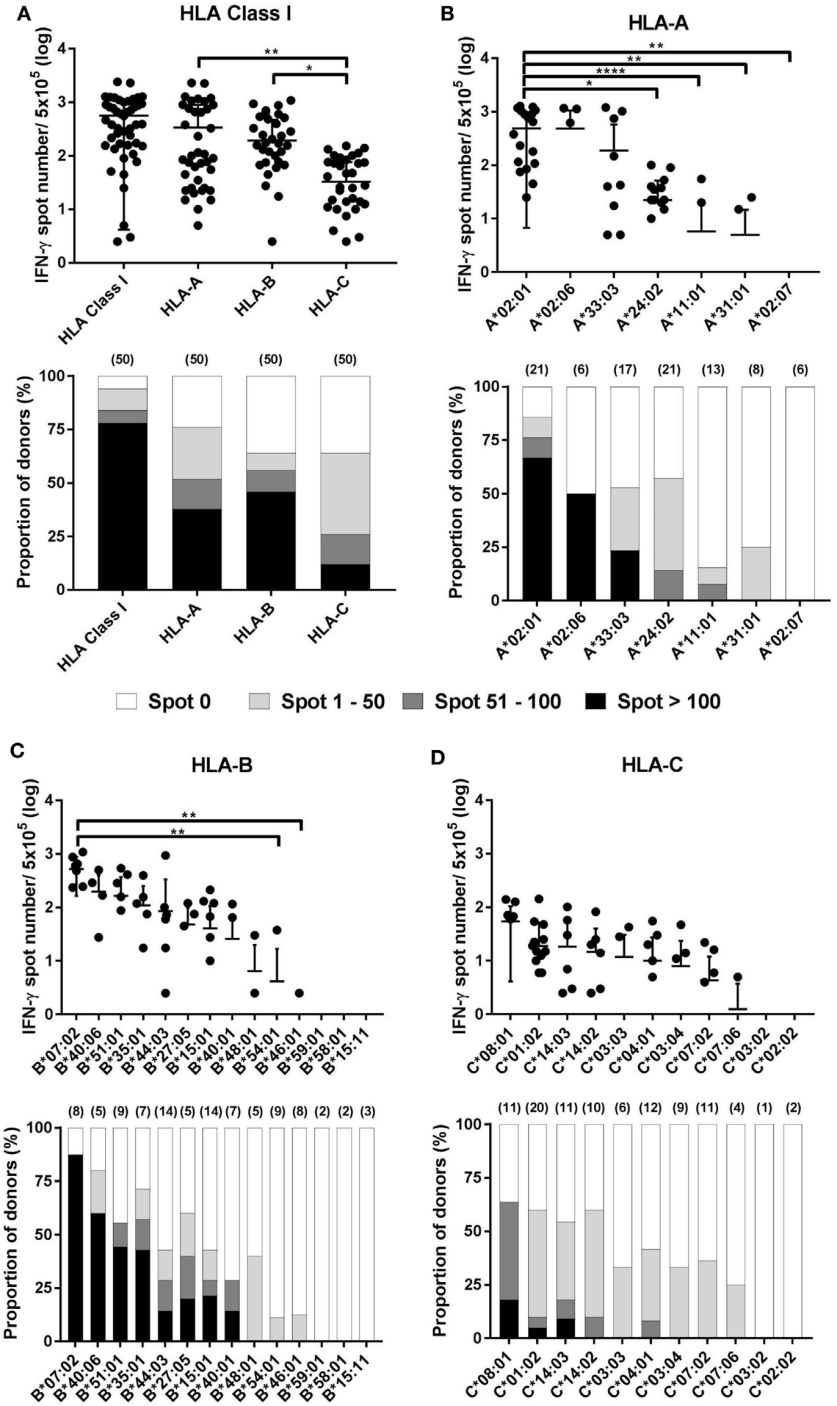
Distribution of cytomegalovirus (CMV) pp65-specific CD8^+^ T cells according to individual human leukocyte antigen (HLA) class I loci and allotypes. Distribution of pp65-specific CD8^+^ T cell responses restricted by total HLA class I, HLA-A, HLA-B, and HLA-C loci **(A)** and distributions of those by each allotype of the HLA-A **(B)**, HLA-B **(C)**, and HLA-C **(D)** loci in healthy Korean donors (*n* = 50). The frequencies of CD8^+^ T cells by interferon-γ (IFN-γ) enzyme-linked immunospot (ELISPOT) assay (upper). CD8^+^ T cell frequencies by HLA class I and by HLA-A, -B, or -C loci is the sum of those by the six allotypes present in an individual and the sum of those by the two allotypes present in each locus, respectively. The relative proportion of donors according to the strengths of CD8^+^ T cell responses (lower). Parenthesis in lower figure indicates the number of donors. *P* values were calculated by one-way ANOVA (*P < 0.05; **P < 0.01; ***P < 0.001; ****P < 0.0001). Horizontal bars indicate mean and vertical bars indicate SD. Because negative numbers cannot be shown on a logarithmic *Y* axis, 0 or negative values and some down vertical bars are not displayed.

The results of the ELISPOT assay for 50 healthy donors were presented as the frequency of CMV pp65-specific CD8^+^ T cells secreting IFN-γ and the proportion by strength of responsiveness (IFN-γ spot numbers per 5 × 10^5^ = 0, 1–50, 51–100, and >100) based on the all allotypes in HLA class I and allotypes in each HLA class I locus (Figure 3A). The distribution of total responses for all allotypes in HLA class I in an individual showed a mean and SD of 564.0 and 559.9, respectively. The proportion of positive responses showing more than 100 IFN-γ spots was 78%, whereas absence of a specific response (spot = 0) was 6%. HLA-A allotypes showed a mean of 338.3 (SD 556.2). The proportion of positive responses showing more than 100 IFN-γ spots was 38%, whereas absence of a specific response was 24%. HLA-B allotypes showed a mean of 193.1 (SD 275.7). The proportion of positive responses showing more than 100 IFN-γ spots was 46%, whereas absence of a specific response was 36%. HLA-C allotypes showed a mean of 32.68 (SD 43.37). The proportion of positive responses showing more than 100 IFN-γ spots was 12%, whereas absence of a specific response was 36%. In the analysis based on each HLA class I locus, the IFN-γ spot numbers in the HLA-A and -B allotypes were significantly higher compared to those in the HLA-C allotypes. The proportion of positive response with more than 100 IFN-γ spots per well was higher in HLA-B than in HLA-A.

In the analysis based on each allotype in the HLA-A locus, the IFN-γ spot number was high in the order HLA-A*02:01 > -A*02:06 > -A*33:03 > -A*24:02 > -A*11:01 (Figure 3B). HLA-A*02:01 allotypes showed a mean of 486.5 (SD 479.8), which was significantly higher compared than those of HLA-A*24:02, -A*11:01, -A*31:01, and -A*02:07. HLA-A*02:01, -A*02:06, and -A*33:03 showed 66.7, 50.0, and 23.5% positive responses with more than 100 IFN-γ spots per well, respectively. In the analysis based on each allotype in the HLA-B locus, the IFN-γ spot number was high in the order HLA-B*07:02 > -B*40:06 > -B*51:01 > -B*35:01 > -B*44:03 > -B*27:05 > -B*15:01 > -B*40:01 (Figure 3C). HLA-B*07:02 showed a mean of 521.9 (SD 358.3), which was significantly higher than those of HLA-B*54:01 and -B*46:01. HLA-B*07:02, -B*40:06, -B*51:01, -B*35:01, -B*15:01, -B*27:05, -B*44:03, and-B*40:01 showed 87.5, 60.0, 44.4, 42.9, 21.4, 20.0, 14.3, and 14.3% positive responses with more than 100 IFN-γ spots per well, respectively. Particularly, HLA-B*07:02 showed the highest proportion of positive responses of donors in all allotypes (7/8, 87.5%), demonstrating more than 100 IFN-γ spots per well in all positive responses. In an analysis based on each allotype in the HLA-C locus, the IFN-γ spot number was high in the order HLA-C*08:01 > -C*01:02 > -C*14:03 > -C*14:02 (Figure 3D). HLA-C*08:01, -C*14:03, and -C*01:02 showed 18.2, 9.1, and 5% positive responses with more than 100 IFN-γ spots per well, respectively. Six allotypes (HLA-A*02:07, -B*59:01, -B*58:01, -B*15:11, -C*03:02, and -C*02:02) showed no positive response.

### Correlation between Positive Reactions of Specific HLA Class I Allotypes and Allele Frequencies

**Figure 4:**
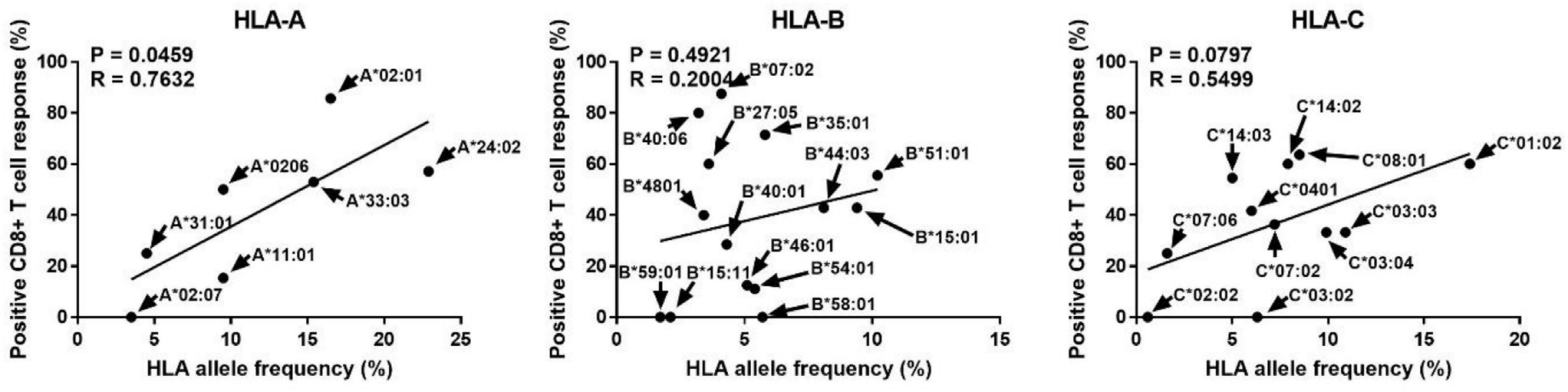
Correlation analysis of the proportion showing positive pp65-specific CD8^+^ T cell responses in each human leukocyte antigen (HLA) allotype with the frequencies of HLA class I alleles known in the Korean population. In the 50 donors included in this study (Table 1), in total 32 allotypes of HLA class I (7 HLA-A, 14 HLA-B, and 11 HLA-C) found. The proportions showing positive CD8^+^ T cell responses in donors with a specific allotype are presented in Figure 3. HLA allele frequencies in the Korean population have been quoted (22). *P* and *R* values calculated by Pearson’s correlation analysis.

Human leukocyte antigen alleles increased in diversity in response to infectious diseases, and people with allotypes responsive to certain infectious agents are known to have better survival and are likely to be evolutionarily selected (20, 21). Therefore, we analyzed whether the allotype restricting CD8^+^ T cell reactivity to pp65, the major antigen of CMV, is related to the frequency of its allele (Figure 4). Interestingly, the ratio of positive CD8^+^ T cell responses in a specific allotype was significantly correlated with the frequency of HLA-A alleles (*P* = 0.0459, *R* = 0.7632). However, HLA-B and HLA-C showed no significant correlation. It is thought that the allele frequency is relatively evenly distributed in HLA-B allotypes and that reactivity to pp65 is low in HLA-C allotypes.

### Intraindividual Dominance and HLA Allotype Hierarchy

**Figure 5:**
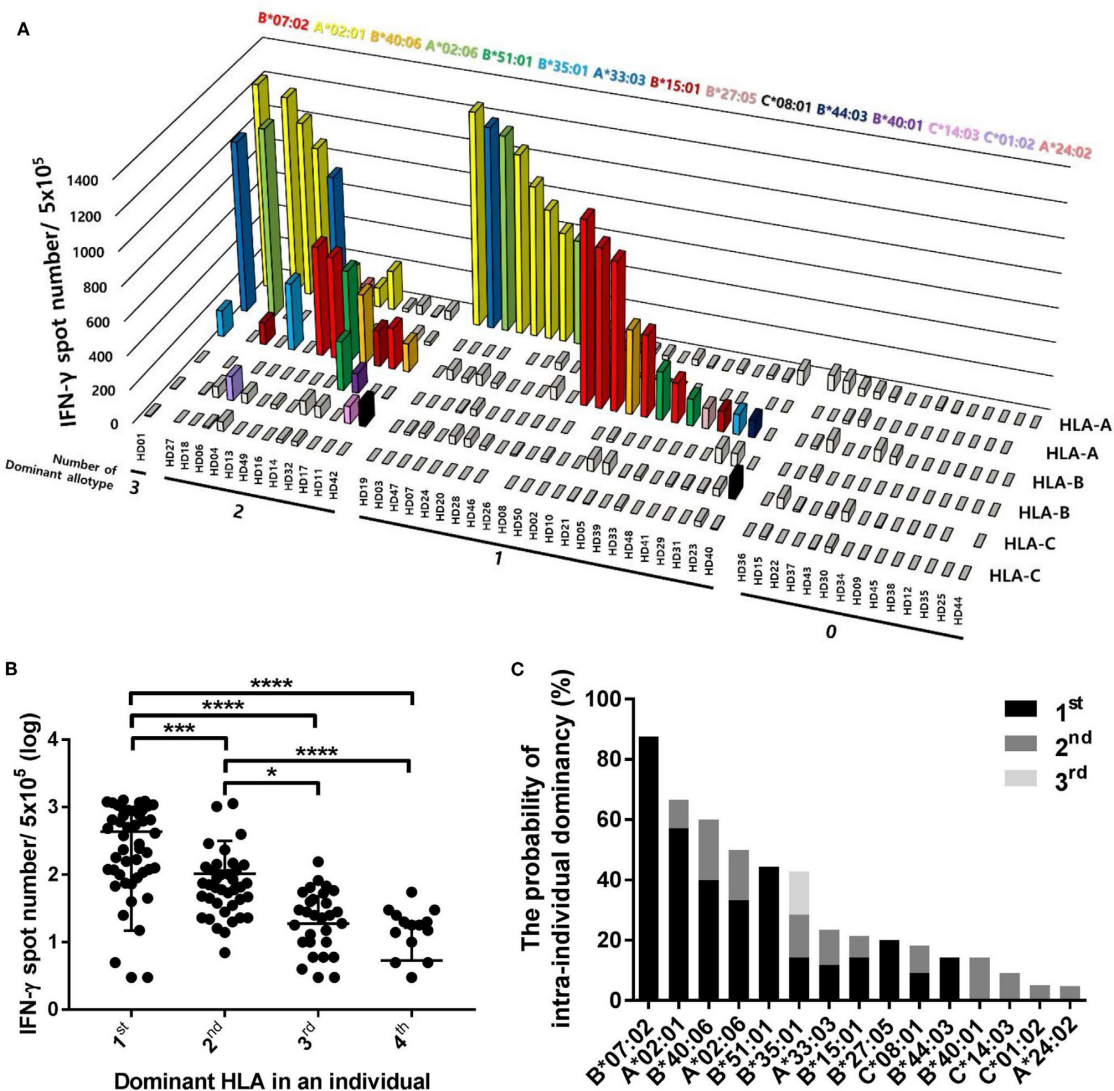
Distributions of CD8^+^ T cell responses according to the intraindividual dominance of human leukocyte antigen (HLA) class I allotypes and the probability of intraindividual dominance in each HLA class I allotype. **(A)** The intraindividual dominant patterns in 50 donors could be classified into four groups by the number of allotypes showing more than 100 interferon-γ (IFN-γ) positive spots per 5 × 10^5^ CD8^+^ T cells in an individual on the *x*-axis, the frequencies of pp65 antigen-specific CD8^+^ T cells by enzyme-linked immunospot (ELISPOT) on the *y*-axis, and displacing the HLA class I allotypes on the *z*-axis. **(B)** When CD8^+^ T cell responses by each HLA class I allotype in an individual were measured by ELISPOT, the first, second, third, and fourth intraindividual dominance was defined in the order of highest response. The distributions of CD8^+^ T cell responses in 50 donors were analyzed according to the intraindividual dominance and **(C)** the probability of intraindividual dominance in each HLA class I allotype was defined in 50 donors with allotypes showing IFN-γ more than 100 positive spots per 5 × 10^5^ CD8^+^ T cells. *P* values were calculated by one-way ANOVA (*P < 0.05; **P < 0.01; ***P < 0.001; ****P < 0.0001). Horizontal bars indicate mean and vertical bars indicate SD. Because negative numbers cannot be shown on a logarithmic *Y* axis, 0 or negative values and some down vertical bars are not displayed.

Because different CD8^+^ T cell responses to pp65 antigen according to HLA allotypes were observed in the population, we comparatively analyzed the effects of each HLA allotype on CD8^+^ T cell responses within an individual to further interpret the population results in terms of the intraindividual dominance and HLA allotype hierarchy. The 50 donors were classified into four groups according to the number of dominant allotypes showing positive responses with more than 100 IFN-γ spots per well in an individual (Figure 5A). There was 1 donor in the three dominant allotype group (2%), 12 donors in the two dominant allotype group (24%), 23 donors in the one dominant allotype group (46%), and 14 donors in the group without a dominant allotype (28%). The one dominant allotype group showed specific responses restricted by 11 allotypes (HLA-B*07:02, -A*02:01, -B*40:06, -A*02:06, -B*51:01, -B*35:01, -A*33:03, -B*15:01, -B*27:05, -C*08:01, and -B*44:03). The two dominant allotype group showed specific responses restricted by 13 allotypes (HLA-B*07:02, -A*02:01, -B*40:06, -A*02:06, -B*51:01, -B*35:01, -A*33:03, -B*15:01, -C*08:01, -B*40:01, -C*14:03, -C*01:02, and -A*24:02). The three dominant allotype group showed specific responses restricted by 3 allotypes (HLA-A*02:01, -B*35:01, and -A*33:03).

We classified the dominance of HLA allotypes into groups 1–4 in the order of the pp65-specific CD8^+^ T cell response in an individual and analyzed the distribution of pp65-specific CD8^+^ T cell responses according to dominance in 50 donors (Figure 5B). The first dominant group (mean of 435.5, SD of 420.6) showed strong significance compared to the second dominant group (mean of 103.7, SD of 214.3, and *P* = 0.0010), which was also significantly higher compared to in the third (mean of 18.78, SD of 29.22, and *P* < 0.0001) and fourth dominant groups (mean of 5.36, SD of 11.1, and *P* < 0.0001).

To define the hierarchy among HLA class I allotypes, the probability of intraindividual dominance was estimated by counting those who showed the first or second dominance in donors with a specific allotype (Figure 5C). HLA-B*07:02, -A*02:01, -B*51:01, -B*40:06, and -A*02:06 exhibited first dominance at 87.5, 57.1, 44.4, 40.0, and 33.3%, respectively. HLA-B*40:06, -A*02:06, -B*35:01, -B*40:01, and -A*33:03 exhibited second dominance at 20.0, 16.7, 14.3, 14.3, and 11.8%, respectively.

### Prediction of Low-Responder to pp65 by HLA Class I Genotype

**Figure 6:**
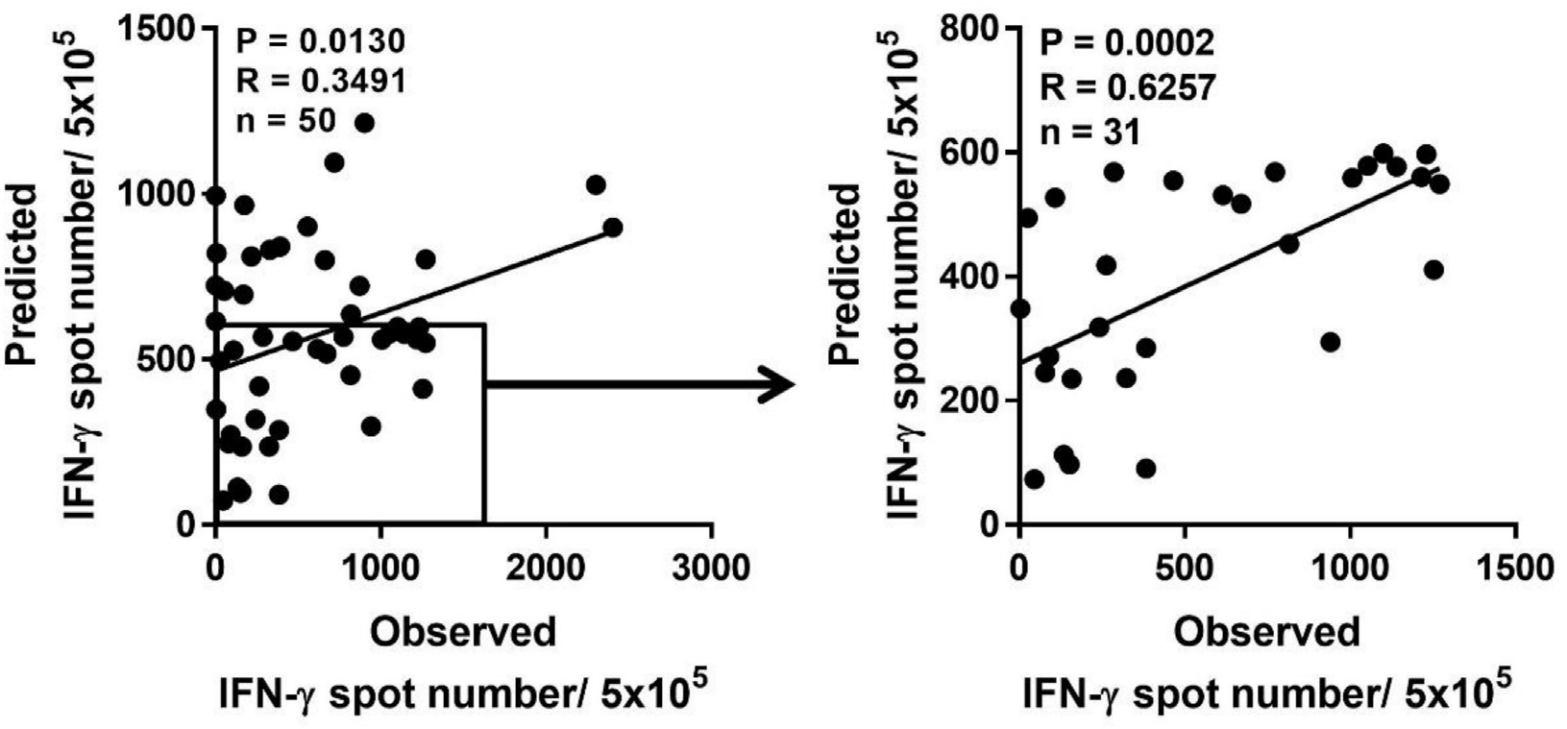
Prediction of pp65-specific CD8^+^ T cell responses in a certain individual based on the database of pp65-specific CD8^+^ T cell responses in each human leukocyte antigen (HLA) class I allotype. Based on the mean frequency of pp65-specific CD8^+^ T cells in each HLA class I allotypes obtained from 50 healthy donors, the sum of CD8^+^ T cell responses in each HLA class I allotype present in an individual was calculated as the predicted CD8^+^ T cell response, which was compared with the observed CD8^+^ T cell response. The predicted CD8^+^ T cell response was the most significant when the response was below 600. *P* and *R* values were calculated by Pearson’s correlation analysis.

Because a low immune response to CMV in donors is associated with the onset of CMV infection in hematopoietic stem cell transplantation, predicting the immune responsiveness to CMV, particularly low immune responsiveness, may be helpful for selecting a suitable donor. Therefore, we estimated the predicted CD8^+^ T cell responsiveness in an individual using the mean frequency of pp65-specific CD8^+^ T cell responses defined in each HLA allotype and compared this value with the observed CD8^+^ T cell response (Figure 6). The correlation between observed and predicted responses for all 50 individuals showed a distinct quantitative linear relationship (*P* = 0.0130, *R* = 0.3491). Because there were some dominant allotypes, the predicted response from all 50 individuals showed a less significant correlation with observed response, and thus we analyzed whether there were more significant correlations in the groups with various low predicted responses. We found a more significant correlation in the group with a predicted value of less than 600 spots/5 × 10^5^ (*P* = 0.0002, *R* = 0.6257; Table 2). These results suggest that donors with low CMVpp65-specific CD8^+^ T cell responses can be predicted by HLA type alone.

**Table 2:** Correlation between the observed responses and the predicted responses according to the prediction ranges.

Prediction range (spots)	Predicted donor (*n*)	*R*	*P*
0–1,200	50	0.3491	0.0130
0–1,100	49	0.3410	0.0165
0–1,000	47	0.2548	0.0839
0–900	44	0.3674	0.0142
0–800	38	0.3793	0.0189
0–700	34	0.4985	0.0027
0–600	31	0.6257	0.0002
0–500	18	0.3174	0.1994
0–400	14	0.1023	0.7279
0–300	11	0.4405	0.1751
0–200	5	0.1387	0.8239
0–100	4	0.3722	0.6278

## Discussion

To determine whether individual HLA class I allotypes are used preferentially or equally in human virus-specific CTL responses, we established a platform for measuring the CD8^+^ T cell response by presenting the pp65 peptide intrinsically to various HLA allotypes using K562-based aAPCs (Figure 1). The coverage of 32 HLA class I allotypes (7 HLA-A, 14 HLA-B, and 11 HLA-C) observed in 50 healthy Korean donors was 85.82% for the HLA-A locus, 75.69% for the HLA-B locus, and 83.04% for the HLA-C locus in the Korean population analyzed in our laboratory data from over 10,000 donors (data not shown). The mean frequency of pp65-specific CD8^+^ T cells was 0.11%. The frequency of CTLs in healthy CMV-seropositive individuals ranged from approximately 0.1 to 3.3% of all CD8^+^ T cells (23).

When the relative contributions of the HLA-A, -B, and -C allotypes were evaluated, immune responses to pp65 in the HLA-C allotypes were lower than the responses to those in the HLA-A and -B loci (Figure 3A). However, there was no difference between the HLA-A and -B allotypes in this study. In general, HLA-C allotypes are expressed on the cell surface at levels much lower than either HLA-A or -B allotypes (24). In this study, the expression of an HLA-C gene was confirmed to be lower than that of HLA-A and -B, although the gene was transferred to the same vector (data not shown). The major role of HLA-C is predicted to be as a ligand for killer immunoglobulin receptors expressed on natural killer cells (25). In human immunodeficiency virus (HIV)-1-infected study subjects from southern Africa, a significantly greater number of CD8^+^ T cell responses are HLA-B-restricted compared to HLA-A (26). This dominant influence of HLA-B may mediate the potential coevolution of HIV and HLA. In a study of EBV-derived epitopes, HLA-B-restricted epitopes were more frequently recognized than HLA-A- or HLA-C-restricted epitopes (14). One possible mechanism underlying the dominant HLA-B allotype effect is the greater diversity of peptides bound by HLA-B allotypes (27).

Human leukocyte antigen-A*02:01, -B*07:02, and -C*08:01 showed the highest magnitude and frequency of immune response to pp65 at each locus (Figures 3B–D). However, HLA-A*02:07, -B*59:01, -B*58:01, -B*15:11, -C*03:02, and -C*02:02 did not show immunoreactivity to pp65. In a study of the contribution of individual HLA-A and -B allotypes to the human influenza virus-specific CTL response, HLA-B*27:05- and HLA-B*35:01-allotypes were preferentially used in the influenza A virus-specific CTL response, whereas the contribution of HLA-B*08:01 and HLA-A*01:01 was minor (28). In contrast, the CTL response to influenza B virus was mainly directed toward HLA-B*08:01-restricted epitopes. Immunodominant HIV-1-specific CD8^+^ T cell responses were defined in both acute and early infection for HLA-A*1, -A*2, -A*24, -B*8, -B*15, -B*40, -B*44, -B*53, -B*57, and -Cw*8 (29). These studies also suggest the preferential use of HLA allotypes according to the virus studied.

Although one individual has up to six different HLA allotypes, 46% of all donors had one dominant allotype, 24% had two dominant allotypes, and 2% had three dominant allotypes presenting pp65 to activate CD8^+^ T cells (Figure 5A). We found that one or two allotypes showed immunodominance, despite the presence of multiple immunodominant allotypes in one individual. CMV-specific cellular immune responses restricted by HLA-B*07 dominate those restricted by HLA-A*02 both in immune-competent and immune-compromised individuals (13). Similar results were observed in HD10 and HD16 in this study. HD10 exhibited an immune response only against HLA-B*07:02, whereas HD16 showed 5.24-fold higher immune response than that of HLA-A*02:01 in HLA-B*07:02. In another case, HLA-B*15:01 showed no dominant response when HLA-A*02:01 was present. HIV-1-specific CD8^+^ T cell responses directed against HLA-A1-, -A2-, and -A24-restricted CD8^+^ T cell epitopes were significantly lower in participants coexpressing HLA-B*57 or HLA-B*27 (29). Certain HLA allotypes consistently contributed more than others to the total virus-specific CD8^+^ T cell response during primary infection and reduced the absolute magnitude of responses restricted by other allotypes if coexpressed in the same individual, which is consistent with immunodominance.

Interestingly, the frequencies of HLA-A alleles were significantly correlated with the positivity of a specific allotype (Figure 4). The significance of HLA class I polymorphisms is that the differences among various HLA molecules and peptides that they present are of sufficient functional importance to be subject to Darwinian natural selection (20). Given the frequency-dependent conservation of HLA, populations in which specific HLA allotypes are very common (e.g., HLA-A*23:01 in West Africa) may be identified as hot spots for severe disease, endemic persistence, or pathogen emergence (21, 30). Thus, a proper understanding of virus-HLA coevolution is instrumental for making informed treatment and vaccine design decisions for a range of major chronic and acute infections (21). Based on our basic data showing the existence of dominance according to HLA allotype, we predicted immune responses to pp65 according to the donor HLA allotype. The observed low responses to pp65 in donors with more than one dominant allotype such as HLA-A*02:01 and/or -B*07:02, who showed nearly the highest predicted responses, reduced the predictive power because of the broad distributions of predicted responses. However, donors without dominant allotype such as HLA-A*02:07, -B*59:01, -B*58:01, -B*15:11, -C*03:02, and/or -C*02:02, showed more accurate prediction because of the narrow distributions of the predicted responses. As a result, more significance was obtained in subjects who had less than a total of 600 counts (Figure 6). Therefore, our results suggest that the HLA allotype as well as direct measurement of T cell responses to CMV may help predict the immune responsiveness to CMV.

Adoptive transfer of partially HLA-matched virus-specific T cells from healthy third-party donors has also shown promise for the treatment of these conditions and strikingly low incidences of toxicity or graft-versus-host disease have been recorded in initial trials (31–33). In this third-party CTL therapy, measurement of T cell responses by specific HLA allotypes may be informative and helpful for partially matched recipient-donors. In addition to the CD8^+^ T cell response against pp65, the CD4 T cell response by a single HLA class II allotype should be further investigated.

In conclusion, our results demonstrate that specific HLA class I allotypes are preferentially used in the CD8^+^ T cell immune response to pp65 and that hierarchy among HLA class I allotypes is present in an individual. This immunodominance improves the understanding of coevolution between CMV infection and HLA diversity. Our strategy of measuring the immune response restricted by a single HLA allotype may predict T cell responsiveness in an individual with known HLA genotypes and provide useful information for vaccine development and third-party CTL therapy for partially matched recipient–donors.

## Ethics Statement

This study was carried out in accordance with the recommendations of institutional guidelines and was approved by Institutional Review Board of the Catholic University of Korea (MC16SNSI0001). Informed consent was obtained according to the Catholic University of Korea. Written informed consent was obtained from all participants involved in this study.

## Author Contributions

Conceived and designed the experiments: S-JH, H-JS, and T-GK. Performed the experiments: S-JH and H-JS. Analyzed the data: S-JH, H-JS, and T-GK. Contributed reagents/materials/analysis tools: H-JL, S-DL, SK, D-HS, C-HH, HC, and H-IC. Wrote the article: S-JH, H-JS, and T-GK.

## Conflict of Interest Statement

The authors declare that the research was conducted in the absence of any commercial or financial relationships that could be construed as a potential conflict of interest.
