# Supplementary material for: Comprehensive Analysis of Cytomegalovirus pp65 Antigen-Specific CD8+ T Cell Responses According to Human Leukocyte Antigen Class I Allotypes and Intraindividual Dominance
Source: Front Immunol. 2017 Nov 21;8:1591. doi: 10.3389/fimmu.2017.01591 (PMC5702484; doi:10.3389/fimmu.2017.01591)
Supplement: Supplementary file 2 [file image_2.pdf]

Supplementary Material

Comprehensive Analysis of CMV pp65 Antigen-Specific CD8$^+$ T Cell Responses According to HLA Class I Allotypes and Intra-Individual Dominance

Seung-Joo Hyun[1], Hyun-Jung Sohn[2], Hyun-Joo Lee[2], Seon-Duk Lee[2], Sueon Kim[1], Dae-Hee Sohn[1], Cheol-Hwa Hong[1], Haeyoun Choi[1], Hyun-Il Cho[2,3] and Tai-Gyu Kim[1,2,3*]

[1]Department of Microbiology, College of Medicine, The Catholic University of Korea, Seoul, Korea,
[2]Catholic Hematopoietic Stem Cell Bank, College of Medicine, The Catholic University of Korea, Seoul, Korea,
[3]Cancer Research Institute, College of Medicine, The Catholic University of Korea, Seoul, Korea

*** Correspondence:** Tai-Gyu Kim: kimtg@catholic.ac.kr

1 Supplementary Data

2 Supplementary Figures and Tables

2.1 Supplementary Figures

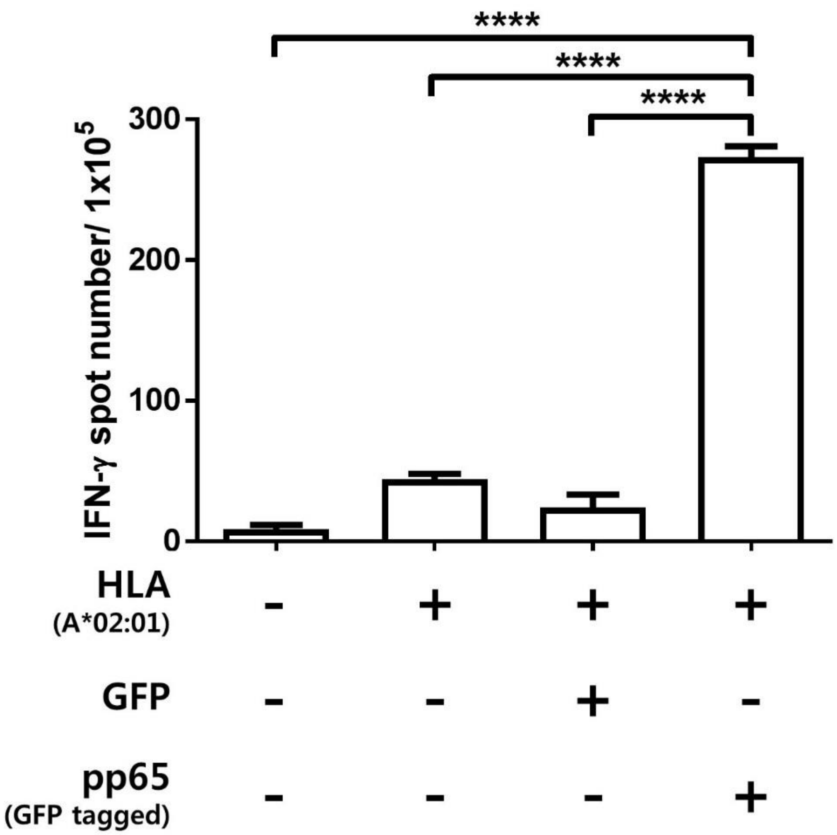

Supplementary Figure 2. Differences in immune responses between GFP and GFP tagged pp65

GFP and GFP-tagged pp65 were used to confirm whether the immune response was caused by GFP. To measure the immune response, the HLA-A*02:01 allotype was transiently expressed in established aAPCs and then GFP and GFP-tagged pp65 were additionally expressed. Immune response was measured by ELISPOT assay and three independent experiments were performed. P values were calculated by 1-way ANOVA (*$P < 0.05$; **$P < 0.01$; ***$P < 0.001$; ****$P < 0.0001$).
