# Supplementary material for: Comprehensive Analysis of Cytomegalovirus pp65 Antigen-Specific CD8+ T Cell Responses According to Human Leukocyte Antigen Class I Allotypes and Intraindividual Dominance
Source: Front Immunol. 2017 Nov 21;8:1591. doi: 10.3389/fimmu.2017.01591 (PMC5702484; doi:10.3389/fimmu.2017.01591)
Supplement: Supplementary file 4 [file image_4.pdf]

Supplementary Material

Comprehensive Analysis of CMV pp65 Antigen-Specific $CD8^+$ T Cell Responses According to HLA Class I Allotypes and Intra-Individual Dominance

Seung-Joo Hyun[1], Hyun-Jung Sohn[2], Hyun-Joo Lee[2], Seon-Duk Lee[2], Sueon Kim[1], Dae-Hee Sohn[1], Cheol-Hwa Hong[1], Haeyoun Choi[1], Hyun-Il Cho[2,3] and Tai-Gyu Kim[1,2,3*]

[1]Department of Microbiology, College of Medicine, The Catholic University of Korea, Seoul, Korea,
[2]Catholic Hematopoietic Stem Cell Bank, College of Medicine, The Catholic University of Korea, Seoul, Korea,
[3]Cancer Research Institute, College of Medicine, The Catholic University of Korea, Seoul, Korea

*** Correspondence:** Tai-Gyu Kim: kimtg@catholic.ac.kr

1 Supplementary Data

2 Supplementary Figures and Tables

2.1 Supplementary Figures

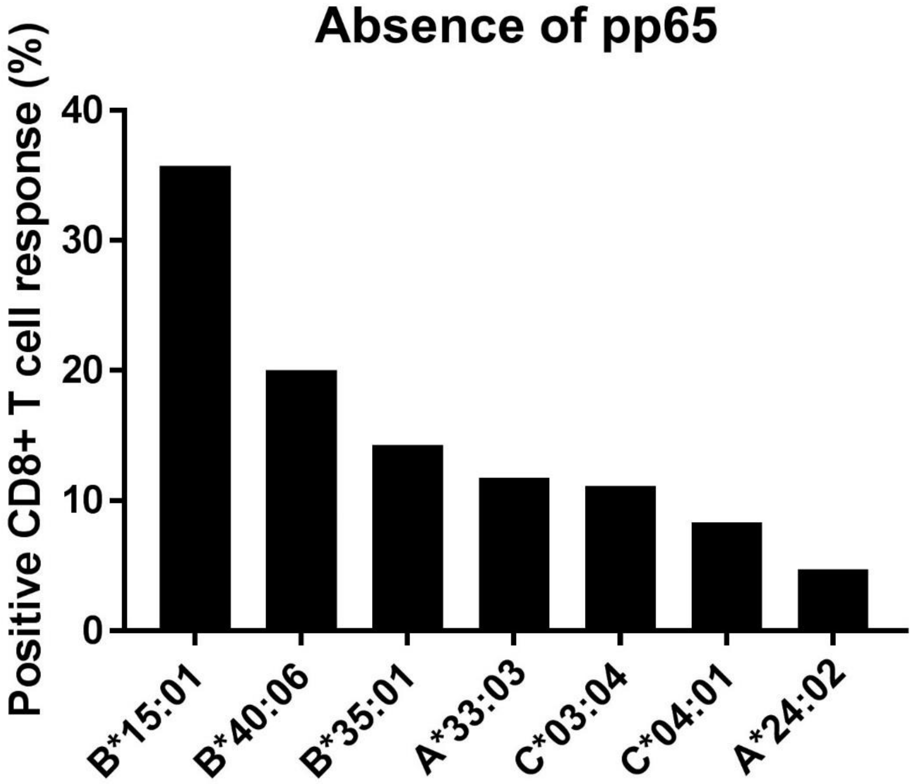

Supplementary Figure 4. HLA-specific CD8$^+$ T cell response without pp65

Specific CD8$^+$ T cell responses were observed in 7 HLA allotypes without pp65. Seven allotypes show strong immune responses more than 100 spots / 5 × 10^5 CD8$^+$ T cells, among them, three allotypes in HLA-B, and two allotypes in HLA-A and –C, respectively. HLA-B*15:01 showed a strong positive CD8$^+$ T cell response of more than 35%.
